# Molecular Identification and Quantification of Tetracycline and Erythromycin Resistance Genes in Spanish and Italian Retail Cheeses

**DOI:** 10.1155/2014/746859

**Published:** 2014-09-11

**Authors:** Ana Belén Flórez, Ángel Alegría, Franca Rossi, Susana Delgado, Giovanna E. Felis, Sandra Torriani, Baltasar Mayo

**Affiliations:** ^1^Departamento de Microbiología y Bioquímica, Instituto de Productos Lácteos de Asturias (IPLA-CSIC), Paseo Río Linares s/n, Villaviciosa, 33300 Asturias, Spain; ^2^Dipartimento di Biotecnologie, Università Degli Studi di Verona, Strada Le Grazie, 15, 37134 Verona, Italy

## Abstract

Large antibiotic resistance gene pools in the microbiota of foods may ultimately pose a risk for human health. This study reports the identification and quantification of tetracycline- and erythromycin-resistant populations, resistance genes, and gene diversity in traditional Spanish and Italian cheeses, via culturing, conventional PCR, real-time quantitative PCR (qPCR), and denaturing gradient gel electrophoresis (DGGE). The numbers of resistant bacteria varied widely among the antibiotics and the different cheese varieties; in some cheeses, all the bacterial populations seemed to be resistant. Up to eight antibiotic resistance genes were sought by gene-specific PCR, six with respect to tetracycline, that is, *tet*(K), *tet*(L), *tet*(M), *tet*(O), *tet*(S), and *tet*(W), and two with respect to erythromycin, that is, *erm*(B) and *erm*(F). The most common resistance genes in the analysed cheeses were *tet*(S), *tet*(W), *tet*(M), and *erm*(B). The copy numbers of these genes, as quantified by qPCR, ranged widely between cheeses (from 4.94 to 10.18log⁡_10_/g). DGGE analysis revealed distinct banding profiles and two polymorphic nucleotide positions for *tet*(W)-carrying cheeses, though the similarity of the sequences suggests this *tet*(W) to have a monophyletic origin. Traditional cheeses would therefore appear to act as reservoirs for large numbers of many types of antibiotic resistance determinants.

## 1. Introduction

Antibiotic resistance (AR) is a natural phenomenon, the appearance of which predates the clinical use of antibiotics [1, 2]. Unfortunately, the widespread use and misuse of antibiotics in clinical and nonclinical environments for more than seven decades have provided optimal conditions for the appearance, mobilization, and concentration of highly efficient resistance systems in bacteria [3]. The transfer of AR genes into human and animal pathogens could ultimately lead to a failure of antibiotic therapy [4]. Mobilization among bacterial species is facilitated by AR genes being commonly located on mobile genetic elements such as transposons and plasmids, which have high horizontal transfer capacity [5]. The presence of antibiotics in the environment not only provides a positive selection for resistant pathogens, but also exerts an evolutive pressure on components of the commensal microbiota [6]. Under these conditions, the commensal bacteria in food could become a reservoir for AR determinants that could then further be disseminated via the food chain [7–9].

Determining the prevalence of AR genes in a given environment, and their characterization, requires the isolation and identification of the resistant bacteria, followed by a molecular analysis of their AR determinants [10–12]. The identification and quantification of AR genes directly in environmental samples, that is, without culturing biases, would be useful [13, 14]. Culture-independent analysis is also faster and more accurate than culture-based methods. Indeed, several AR identification and AR gene quantification techniques that require no culturing have recently been developed, such as denaturing gradient gel electrophoresis (DGGE) [15, 16], real-time quantitative PCR (qPCR) [13, 14, 17], construction and functional analysis of gene libraries [18–20], AR gene microarrays [21, 22], and analysis of metagenomic sequences [23, 24]. Knowledge of the types and loads of AR genes in foods could ultimately be of help to estimate the risk of their transmission during cheese manufacturing and after consumption. To our knowledge, few attempts have been made to directly analyze AR gene numbers and diversity in food samples.

The aims of the present work were to identify the microbial populations resistant to tetracycline and erythromycin in commercial Spanish and Italian cheeses and to quantify their antibiotic resistance genes, using culturing and molecular techniques. These antibiotics were selected as a model due to the fact that resistance against tetracycline and erythromycin has been extensively documented among food-borne bacteria, including strains of lactic acid bacteria species.

## 2. Material and Methods

### 2.1. Cheese Sampling

Twenty commercial cheeses—10 Spanish (Cabrales, Zamorano, Majorero, Mahón, Torta del Casar, Manchego, Ibores, Garrotxa, De La Mesta, and Ibérico) and 10 Italian (Gorgonzola “dolce,” Gorgonzola “picante,” Caprino, Quartirolo Lombardo, Pecorino Sardo, Grana Padano, Montasio, Monte Veronese, Asiago, and Taleggio)—were bought at retail stores. These cheese types vary in terms of the technology used to manufacture them (artisanal (without starters), industrial (using commercial starters), the type of milk used (cow, goat, ewe, or mixtures), the treatment to which that milk is subject (raw, pasteurised), and ripening time (from one to 24 months)).

### 2.2. Plate Counts of Cultivable Antibiotic-Resistant Bacteria

Cubes of ten grams of cheese from the centre were homogenised with 90 mL of a 2% (w/v) sterilised sodium citrate solution prewarmed at 45°C for 1 min in a Colworth Stomacher 400 (Seward Ltd., London, UK). Cheese homogenates were tenfold diluted in Ringer's solution (Merck, Darmstadt, Germany) and the dilutions were plated in duplicate on selective and nonselective agarified media. Total aerobic mesophilic bacteria (including aerotolerant and facultative anaerobes) were enumerated on Plate Count Milk Agar (PCMA; Merck), Gram-positive bacteria such as lactic acid bacteria (LAB) were enumerated using de Man Rogosa and Sharpe agar (MRS; Merck), enterococci on Slanetz and Bartley agar (SB; Merck), and staphylococci on Baird-Parker agar (BP; Merck). Tetracycline and erythromycin resistant populations were counted using the same nonselective selective and media containing tetracycline (15 *μ*g mL^−1^) and erythromycin (8 *μ*g mL^−1^) (Sigma-Aldrich, St. Louis, MO, USA). Antibiotic concentrations were considered eight- to tenfolds higher than the usual resistance levels of susceptible populations [29]. This concentration, which is much lower than that provided by dedicated resistance mechanisms [5, 29], was considered to avoid unspecific growth of susceptible bacteria. Plates were then incubated at 30°C (PCMA and MRS media) and 45°C (SB medium) for 72 h and at 37°C (BP medium) for 48 h.

### 2.3. Total DNA Extraction and Purification

Cheese samples (5 grams) were homogenized with 45 mL of a 2% sterile sodium citrate solution and incubated at 37°C for 3 h in the presence of 1 mg mL^−1^ pronase (Sigma-Aldrich) and 100 *μ*L *β*-mercaptoethanol (Merck). Total microbial cells were harvested by centrifugation at 5000 ×g for 20 min and disrupted using 0.1 mm glass beads (Sigma-Aldrich) as reported elsewhere [30]. Genomic DNA was purified by phenol and phenol/chloroform extractions and precipitated with 2-propanol (all chemicals from Sigma-Aldrich). Finally, it was suspended in sterile water containing 5–15 mg mL^−1^ of RNase (Sigma-Aldrich).

Total DNA was also isolated from pure cultures of control strains carrying known AR genes:* Staphylococcus epidermidis* SE36 [*tet*(K)],* Enterococcus faecium* ET51 [*tet*(L)],* Lactococcus lactis* IPLA 31008 [*tet*(M)],* Enterococcus faecalis* Jtet [*tet*(O)],* Enterococcus* spp. ET15 [*tet*(S)],* Bifidobacterium longum* B93 [*tet*(W)],* Lactobacillus johnsonii* G41 [*erm*(B)], and* Bacteroides fragilis* 79a [*erm*(F)]. Genomic microbial DNA from these strains was purified from 1 mL of an overnight culture in brain heart infusion broth (BHI; Merck) using the Kit GenElute bacterial genomic DNA (Sigma-Aldrich). The recovered DNA was then stored at −20°C until analysis.

### 2.4. PCR Detection of Tetracycline and Erythromycin Resistance Genes

The presence of tetracycline and erythromycin resistance genes was examined in DNA from the cheese samples and from control strains by standard PCR using both universal primers for genes encoding tetracycline resistance through ribosomal protection proteins (RPPs), such as* tet*(M),* tet*(O),* tet*(S), and* tet*(W), and gene-specific primer pairs for genes encoding tetracycline resistance [*tet*(K) and* tet*(L)] and erythromycin resistance [*erm*(B) and* erm*(F)] (Table 1). The PCR conditions used for amplification were those reported in Table 1. Positive (DNA from appropriate control strains) and negative (no template DNA) controls were subjected to amplification under the same conditions.

### 2.5. Real-Time Quantitative PCR

#### 2.5.1. Design of Primers

Updated tetracycline and erythromycin resistance genes in databases were compiled for designing of new primers for the qPCR analysis. Nucleotide sequences encoding tetracycline resistance in the form of RPPs and efflux pumps, and erythromycin resistance, were downloaded from the GenBank database (see Table 1 in Supplementary Material available online at http://dx.doi.org/10.1155/2014/746859) and aligned with one another using Mega 5 software. Conserved regions at appropriate distances were used for designing the primers employed in qPCR reactions (Table 1). This was achieved using Primer Express 3.0 software (Applied Biosystems, Carlsbad, CA, USA).

#### 2.5.2. qPCR Conditions

qPCR analyses were performed to quantify tetracycline (*tet*) and erythromycin (*erm*) resistance genes in the total microbial DNA from the cheese samples. Amplifications were performed in an ABI Prism Fast 7500 sequence detection system (Applied Biosystems). Each 20 *μ*L qPCR reaction included 4 *μ*L of extracted DNA (approximately 200 ng), 10 *μ*L of SYBR Green PCR Master Mix (containing ROX as a passive reference), and 900 nM of each primer. The following cycling conditions were used: 2 min at 50°C, an initial denaturation step of 10 min at 95°C, followed by 40 cycles of 15 s at 95°C, and 60 s at 60°C. Baseline and threshold calculations were performed using ABI Prism Fast 7500 software. Melting temperature analyses and size estimations of the PCR products were performed on agarose gels to check for nonspecific amplification.

#### 2.5.3. Construction of qPCR Standards

Conventional PCR reactions, as described above, were used to generate gene-specific amplicons used as template DNA standards for qPCR. Amplicons were purified using a gel/PCR extraction kit (ATP Biotech, Banciao City, Taiwan) and quantified fluorometrically using a Gen5 Microplate dsDNA Quantitation Kit (Biotek Instruments, Winooski, VT, USA). The number of DNA molecules was calculated based on the size and mass of the amplicons. Tenfold serial dilutions of the amplicons were prepared and used following the procedure by Yu et al. [17]. To ensure accuracy in copy number quantification, a complementary control standard curve was obtained using DNA from known numbers of* B. longum* B93 cells (enumerated in MRS agar plates containing 0.25% cysteine following incubation in anaerobiosis at 37°C for 72 h); this strain carries a single copy of* tet*(W) [31]. Dilutions of total DNA from the cells were subjected to qPCR analysis as above.

#### 2.5.4. qPCR Expression Data

Absolute gene copy number was expressed as the number of copies of resistant genes per g^−1^, whereas relative copy number was expressed as the number of resistant genes per million copies of total bacteria ribosomal genes, as determined with the universal prokaryotic primers TBA-F and TBA-R (Table 1). Absolute abundance was calculated based on the results obtained for the corresponding standard of each resistant gene. Relative abundance was calculated using *E* ^ΔCt^, where *E* is the efficiency of the primer according to the slope of standard curve (*E* = 10^−1/slope^) and ΔCt is the Ct value of the gene target (tetracycline and erythromycin resistance genes) normalized against the Ct value of the total bacterial numbers in the samples. For the relative quantification, the copy number of the 16S rRNA genes per cell was averaged to five. The efficiency *E* of each pair of primers was as follows: 1.94 for* tet*(K), 1.97 for* tet*(L), 1.97 for* tet*(M), 2,03 for* tet*(O), 2,02 for* tet*(S) and 2,03 for* tet*(W), and 1.94 for* erm*(B) and 1.99 for* erm*(F).

### 2.6. Denaturing Gradient Gel Electrophoresis Analysis

DNA from cheeses and strains harbouring known* tet* genes (*Lactococcus lactis* IPLA 31008 [*tet*(M)],* Enterococcus faecalis* Jtet [*tet*(O)],* Enterococcus* spp. ET15 [*tet*(S)], and* Bifidobacterium longum* B93 [*tet*(W)]) was amplified by PCR using DGGE primers (Table 1), employing the PCR conditions described elsewhere [15]. DGGE analysis of the amplified* tet* genes was performed as previously reported [16] with slight modifications. Briefly, DGGE was performed in a DCode apparatus (Bio-Rad, Richmond, CA, USA) at 60°C on 8% polyacrylamide gels with a formamide-urea denaturing gradient of 15–50%. Electrophoresis was conducted at 150 V for 2 h and 200 V for 1 h. After electrophoresis, gels were stained in an ethidium bromide solution (0.5 *μ*g mL^−1^), and the DNA bands were visualized and captured using a Gbox system and GeneSys software (Syngene, Cambridge, UK). After isolation and reamplification with the same primers without the GC clamp and identical PCR conditions, bands from the acrylamide gels were identified by sequencing and sequence comparison. Online similarity searches were performed by using the BLAST tool in the GenBank database (http://www.ncbi.nlm.nih.gov/BLAST/).

## 3. Results

### 3.1. Prevalence of AR Populations in Spanish and Italian Cheeses

The Spanish and Italian cheese samples were subjected to conventional microbiological analysis on selective and differential media supplemented or not with tetracycline and erythromycin. Populations of tetracycline-resistant (Tet^r^) and/or erythromycin-resistant bacteria (Erm^r^) were detected on most counting media for both Spanish and Italian cheeses made from raw or pasteurised milk (Tables 2 and 3, resp.). The AR bacterial counts varied widely depending on the microbial group and type of cheese. In general, Italian cheeses (Table 3) returned lower Tet^r^ and Erm^r^ counts than Spanish cheeses (Table 2). Table 2 shows the total number of mesophilic, aerobic, Tet^r^, and Erm^r^ bacteria (enumerated in PCMA) to reach a maximum of 6.79 log_10_ cfu g^−1^ and 7.15 log_10_ cfu g^−1^, respectively, in samples of Cabrales. Microbial counts in this cheese type showed the antibiotic-resistant populations to generally be two to three log_10_ units lower than in antibiotic-free media; such results were only returned by this cheese.

### 3.2. Prevalence of AR Genes in Spanish and Italian Cheeses

Table 4 shows the resistance genes identified by PCR in total DNA from the different cheeses using specific primers for six tetracycline and two erythromycin resistance genes. Two or more AR genes were detected in all cheeses with the exception of Mahón and Gorgonzola “dolce” cheeses, both of which returned a single AR gene (*tet*(W) and* tet*(K), resp.). Indeed, the Spanish De La Mesta cheese harboured all eight AR genes examined in this work, while the Italian Asiago cheese harboured seven (all but* tet*(L)). The most common gene encoding tetracycline resistance was* tet*(S), which was identified in nine Spanish and six Italian cheeses, followed by* tet*(W) in seven Spanish and four Italian cheeses. In contrast,* tet*(L) and* tet*(O) were the least widespread of the tetracycline resistance determinants; these were detected in three Spanish cheeses and in two Spanish and two Italian cheeses, respectively. The* erm*(B) gene was detected in all but three cheese types, while the* erm*(F) gene was identified in seven of the 10 Italian cheeses, but only in one Spanish cheese (De La Mesta).

### 3.3. Quantification of Tetracycline and Erythromycin Resistance Genes by qPCR

**Figure 1:**
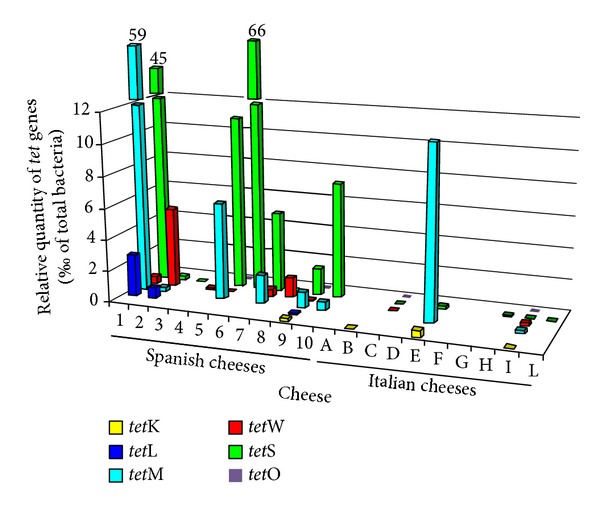
Bars diagram depicting the percentage copy number (‰) of different tetracycline resistance genes quantified by qPCR as a function of the total bacteria in Spanish (1 to 10) and Italian (A to L) cheeses. The order of both Spanish and Italian cheese types is the same as in the tables.

**Figure 2:**
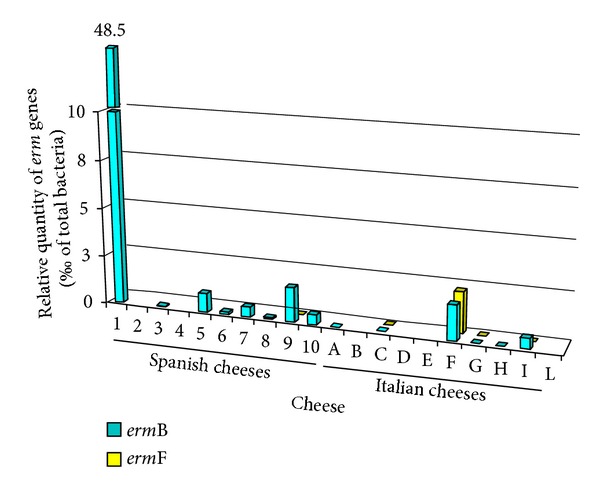
Bars diagram depicting the percentage copy number (‰) of* erm*(B) and* erm*(F) erythromycin resistance genes quantified by qPCR as a function of the total bacteria in Spanish (1 to 10) and Italian (A to L) cheeses. The order of both Spanish and Italian cheese types is the same as in the tables.

Samples positive for AR genes in gene-specific PCR were subjected to qPCR to quantify those identified. A standard curve encompassing 10^3^ through 10^10^ gene copies per reaction was produced using total DNA from* B. longum* B93 as a control (*r* ^2^ ≥ 0.996). The qPCR detection limit determined from both the control DNA of* B. longum* and the serial dilutions of gene-specific amplicons was estimated to be about 10^4^ copies per gram of cheese. Independent reactions were performed in triplicate; high reproducibility was always obtained (average standard deviation ≤0.3). The amplification of AR genes with conventional gene-specific PCR and qPCR primers returned similar results for the Spanish cheeses (Table 4; Figures 1 and 2). Indeed, only two samples showed qPCR amplification failures for genes previously detected by gene-specific PCR among the Spanish cheeses (one* tet*(K) and one* erm*(B) gene). In contrast, among the Italian cheeses, eight samples showing AR genes in gene-specific PCR analysis (three for* erm*(F), two for each* erm*(B) and* tet*(W), plus one for* tet*(S)) failed to show the same with qPCR-specific primers.

The copy number of the different tetracycline (Figure 1) and erythromycin (Figure 2) resistance genes varied widely among the cheese types. Absolute copy numbers of AR genes (log_10_/g of cheese) ranged from 4.94 (*erm*(F) in Asiago cheese) to 10.18 (*tet*(S) in Torta del Casar cheese). On average, the copy number of the different AR genes, quantified by qPCR analysis, was 6.6 ± 1.2 copies per gram of cheese. Figures 1 and 2 show the number of each resistance gene as a percentage (‰) of the total bacterial loads in the cheeses as determined by 16S rDNA analysis. In this way, intersample variations in gene copy number between cheeses are clearly depicted. In general agreement with the microbial counting results, fewer AR gene copies were found in the Italian than in the Spanish cheeses.

### 3.4. Denaturing Gradient Gel Electrophoresis (DGGE) Analysis

**Figure 3:**
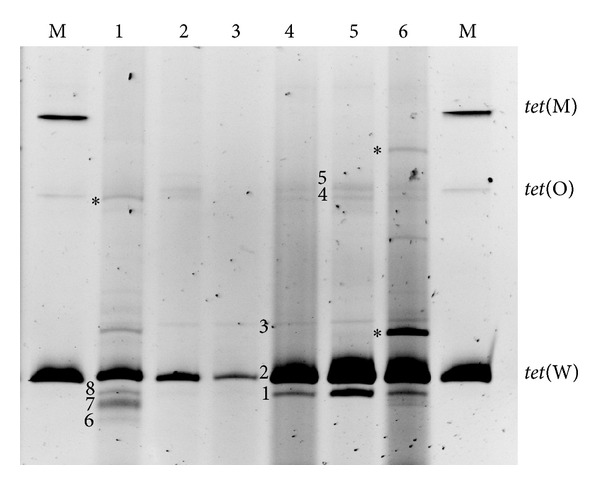
PCR-DGGE analysis of total DNA from Spanish cheeses using specific DGGE-primers for the tetracycline resistance* tet*(W) gene. Lanes from 1 to 6, Cabrales, Zamorano, Torta del Casar, Ibores, Garrotxa, and De La Mesta. Lanes M, DGGE markers consisting of amplicons obtained with specific DGGE primers for* tet*(M),* tet*(O), and* tet*(W), and using as template total DNA from bacterial strains harboring the respective tetracycline resistance gene. The asterisks denote bands that did no reamplification and, consequently, could not be identified.

DGGE analyses were undertaken to assess the intragenic sequence variability of two of the most prevalent AR genes,* tet*(W) and* tet*(M). Besides differences in gene copy number, sequence variability can be a cause of amplification failure in gene-specific PCR and qPCR.  Figure 3 shows the DGGE profiles obtained for* tet*(W) using total DNA from Spanish cheese samples as a template. The DGGE primers returned amplifications in six of the seven cheese samples deemed positive by gene-specific PCR (all but Mahón cheese). A prominent band was present in all samples analysed, the mobility of which was comparable to that produced when using as a template DNA from the* B. longum* control strain carrying a chromosomal* tet*(W) gene. Ten other bands of minor intensity were also observed across the cheeses (up to six bands in one sample; Figure 3, line 1), of which eight could be reamplified and sequenced. Sequence comparison showed these bands to be of identical nucleotide sequence to* tet*(W), except at two polymorphic positions. These polymorphic bands migrated differently on the gel. However, surprisingly, some bands from different positions showed identical sequences.

DGGE analysis was also performed with specific primers for* tet*(M) using DNA from cheeses in which this gene had been identified. In this case, no differences were seen among the DGGE profiles. All the profiles obtained were composed of one intense and two weak bands, but all these bands were also produced when DNA from a control strain carrying a single-copy* tet*(M) gene was used as a template (data not shown).

## 4. Discussion

It is acknowledged that the misuse and overuse of antibiotics in agriculture, aquaculture, animal husbandry, and the clinic have caused an increase in AR bacteria, the consequence of the selection pressure exerted in these environments [3, 32]. AR in dairy products has traditionally been examined by conventional methods of microbial analysis. Indeed, in the present work, the tetracycline and erythromycin resistant populations of 20 Spanish and Italian traditional cheeses were first identified by culturing. High numbers of Tet^r^ and Erm^r^ bacteria were found in most samples, suggesting cheese to be an important vehicle of AR genes within the food chain. Similar ratios between counts of AR and antibiotic susceptible bacteria have previously been reported in other cheese types, such as Mozzarella [33] and Cheddar [8].

No significant differences were seen in AR counts between cheeses made from raw or pasteurised milk or between cheeses made from milk of different species. These results agree with previous reports that indicate reductions in the presence of AR bacteria only in sterile foods [4]. Pasteurisation may reduce the numbers and types of environmental bacteria, but traditional cheesemaking is open to contamination, in which organisms from many sources can gain access to and develop in the cheese. The Italian cheeses contained smaller AR populations than the Spanish ones. The curd-cooking step in some Italian cheeses may reduce the number of nonstarter microorganisms (e.g., enterococci and staphylococci), which have been shown to contain a plethora of resistance types [34–36]. Further, AR counts seemed to be inversely correlated with ripening time (data not shown), which is usually longer for Italian cheeses.

The presence of a wide range of tetracycline and erythromycin resistance genes in microbial populations from different environments has been reported elsewhere [13–15, 37, 38]. In the present study, the most abundant* tet* and* erm* genes were* tet*(S),* tet*(W),* tet*(M), and* erm*(B). In addition,* erm*(F) was widespread among the Italian cheeses.

The detection of* tet* and* erm* resistance genes by qPCR has frequently been used to monitor AR gene loads in different environments [13, 39–41]. However, it has been little used with dairy products [14, 37]. In the present work, great variation was seen between the cheeses in terms of the total abundance of Tet^r^ and Erm^r^ genes. Absolute copy numbers higher than 5 log_10_ units have previously been reported for different* tet* genes [37]. In the present work, gene copy numbers were much higher than the corresponding AR bacterial counts in antibiotic-containing media. Similar results have recently been reported for* tet*(A) and* tet*(B) in hake using qPCR and Taqman probes [42]. This might be a reflection of the actual copy number of the genes in AR bacteria (e.g., they may be present in high copy number plasmids) or due to the fact that DNA-based techniques do not distinguish between live, viable-but-noncultivable, and dead bacteria [13, 14, 37, 42]. Despite this, qPCR results showed a similar trend to plate counting. Lower gene copy numbers and AR bacterial counts were always recorded for the Italian cheeses. Intragenic variability, amplification yields by different primer pairs, and/or the high qPCR detection limit (of 10^4^ copies per gram of cheese) might explain the discrepancies between the results obtained by this technique and those by gene-specific PCR.

As mentioned earlier, in addition to its use for tracking bacterial populations, the DGGE technique has already been used to analyse the polymorphism of tetracycline resistance genes in several environments [15, 16]. However, to our knowledge, this is the first attempt to use this molecular tool to estimate the diversity of* tet* genes in dairy products. A canonical* tet*(W) and* tet*(M) sequence accounted for the majority of genes in all cheeses. Nevertheless, single-nucleotide differences at two positions in the analysed segment of* tet*(W) were noted. Whether polymorphic sequences are carried by different bacterial species remains unknown. Nevertheless, the low sequence divergence suggests that* tet* resistance genes in cheese have a monophyletic origin and are spread among the majority bacterial populations through horizontal transfer. This has already been reported for* tet* genes in bacterial populations from other environments [43, 44].

## 5. Conclusions

The Spanish and Italian cheeses analyzed in this work showed wide variation in their AR bacterial populations, AR gene diversity, and AR gene loads for resistance to tetracycline and erythromycin. The results of this research are of basic and applied interest. Methods and techniques can be extended to study resistance against other groups of antibiotics of currently higher clinical significance, such as *β*-lactams and aminoglycosides. On average, more than three different AR genes were detected in every cheese type. Indeed, all eight* erm* and* tet* resistance genes searched for were identified in one Spanish cheese (De La Mesta) and seven in one Italian cheese (Asiago). The diversity of genes and their large copy numbers can be considered as a biological hazard with a likely, yet undefined, risk of horizontal transfer. To fully assess this risk would require the genetic characteristics of the bacteria carrying these AR genes and the location of these genes in the genome (chromosome, plasmid, transposon, integrons, etc.) to be known. As some other foods of animal origin, cheeses might be key players on the spread of AR genes via the food chain. This is certainly a biological hazard, which anticipates a transfer to susceptible bacteria during cheese manufacture or after consumption. Therefore, improvements in hygiene in animal husbandry, milk production, and cheese manufacturing practices may contribute to preventing the spread of these (and maybe others) AR determinants.

## Supplementary Material

Supplementary Table 1 summarizes updated tetracycline and erythromycin resistance genes which were compiled for designing primers for the qPCR analyses. Nucleotide sequences were downloaded from the GenBank database and aligned with one another using Mega 5 Software.

## Figures and Tables

**Table 1:** Sequence and properties of the PCR primers used in this work.

Application/primer	Target gene	Sequence (5′-3′)	Annealing *T* ^a^ (°C)	Amplicon size (bp)	Reference
Conventional PCR					
Tet-F	*tet* ^ a^	GCTCA(T/C)GTTGA(T/C)GCAGGAA	50	1292	[25]
Tet-R		AGGATTTGGCGG(C/G)ACTTC(G/T)A			
TetK-F	*tet*(K)	TTATGGTGGTTGTAGCTAGAAA	55	348	[15]
TetK-R		AAAGGGTTAGAAACTCTTGAAA			
TetL-F	*tet*(L)	GTMGTTGCGCGCTATATTCC	55	696	[15]
TetL-R		GTGAAMGRWAGCCCACCTAA			
DI-F	*tet*(M)	GAYACICCIGGICAYRTIGAYTT	55	1513	[26]
TetM-R		CACCGAGCAGGGATTTCTCCAC			
TetO-F	*tet*(O)	AATGAAGATTCCGACAATTT	55	781	[26]
TetO-R		CTCATGCGTTGTAGTATTCCA			
TetS-F	*tet*(S)	ATCAAGATATTAAGGAC	55	573	[26]
TetS-R		TTCTCTATGTGGTAATC			
TetW-F	*tet*(W)	AAGCGGCAGTCACTTCCTTCC	50	1150	[11]
Tet-R		AGGATTTGGCGG(C/G)ACTTC(G/T)A			
ErmB-F	*erm*(B)	GAAAAGGTACTCAACCAAATA	50	639	[27]
ErmB-R		AGTAACGGTACTTAAATTGTTTAC			
ErmF-F	*erm*(F)	CGGGTCAGCACTTTACTATTG	50	466	[27]
ErmF-R		GGACCTACCTCATAGACAAG			
Real-time qPCR					
TBA-F	16S rDNA	CGGCAACGAGCGCAACCC	60	130	[28]
TBA-R		CCATTGTAGCACGTGTGTAGCC			
TetK-qPCR-F	*tet*(K)	TGCTGCATTCCCTTCACTGA	60	69	This study
TetK-qPCR-R		GCTTTGCCTTGTTTTTTTCTTGTAA			
TetL-qPCR-F	*tet*(L)	GGGTAAAGCATTTGGTCTTATTGG	60	63	This study
tetL-qPCR-R		ATCGCTGGACCGACTCCTT			
TetM-qPCR-F	*tet*(M)	CAGAATTAGGAAGCGTGGACAA	60	67	This study
TetM-qPCR-R		CCTCTCTGACGTTCTAAAAGCGTAT			
TetO-qPCR-F	*tet*(O)	AATGTCAGAACTGGAACAGGAAGAA	60	59	This study
TetO-qPCR-R		CGTGATAAACGGGAAATAACGTT			
TetS-qPCR-F	*tet*(S)	CGAGGTCATTCTCATTGGTGAA	60	84	This study
TetS-qPCR-R		CAGACACTGCGTCCATTTGTAAA			
TetW-qPCR-F	*tet*(W)	ACGGCAGCGCAAAGAGAA	60	60	This study
TetW-qPCR-R		CGGGTCAGTATCCGCAAGTT			
ErmB-qPCR-F	*erm*(B)	GGATTCTACAAGCGTACCTTGGA	60	69	This study
ErmB-qPCR-R		AATCGAGACTTGAGTGTGCAAGAG			
ErmF-qPCR-F	*erm*(F)	TGATGCCCGAAATGTTCAAGT	60	63	This study
ErmF-qPCR-R		AAAGGAAATTTCGGAACTGCAA			
DGGE assays					
TetM-F	*tet*(M)	ACAGAAAGCTTATTATATAAC	55	171	[15]
GC^b^-TetM-R		TGGCGTGTCTATGATGTTCAC			
TetW-F	*tet*(W)	GAGAGCCTGCTATATGCCAGC	64	168	[15]
GC-TetW-R		GGGCGTATCCACAATGTTAAC			

^a^ *tet*, genes encoding ribosome protection proteins.

^b^Sequence of the GC clamp: CGCCCGGGGCGCGCCCCGGGCGGGGGGGGGGCACGGGGGG.

**Table 2:** Microbial counts (log_10_⁡ cfu g^−1^) of the total and antibiotic resistant bacterial populations found in samples of ten Spanish cheeses.

Culture medium^a^	Cheese									
	Cabrales∗	Zamorano∗	Majorero∗	Mahón	Torta del Casar∗	Manchego	Ibores∗	Garrotxa	De La Mesta∗	Ibérico
PCMA	7.74	6.64	9.23	6.49	8.18	8.21	8.26	7.58	6.01	8.62
PCMA + Tc	6.76	2.74	4.26	<2.0^b^	5.98	3.21	5.76	3.68	5.46	4.70
PCMA + Erm	7.15	3.92	4.36	3.84	5.82	3.16	5.65	4.72	3.91	3.62

MRS	7.20	6.53	4.53	6.36	7.93	6.21	8.12	7.40	5.08	7.92
MRS + Tc	6.79	3.64	4.11	<2.0	4.96	<2.0	4.77	3.12	<2.0	<2.0
MRS + Erm	6.90	4.18	4.03	<2.0	4.89	<2.0	5.67	3.22	<2.0	<2.0

SB	5.83	4.07	2.95	<2.0	5.67	<2.0	5.85	3.13	4.82	5.04
SB + Tc	5.08	2.30	<2.0	<2.0	4.82	<2.0	3.20	<2.0	2.81	4.24
SB + Erm	5.18	3.04	<2.0	<2.0	4.95	<2.0	<2.0	<2.0	3.61	<2.0

BP	5.34	3.53	3.91	3.74	5.30	<2.0	4.27	4.26	4.18	4.72
BP + Tc	4.71	2.78	<2.0	<2.0	4.84	<2.0	2.98	3.20	<2.0	<2.0
BP + Erm	5.00	2.98	3.56	<2.0	4.95	<2.0	<2.0	4.47	<2.0	<2.0

^a^Culture media used for counting of different bacteria groups: PCMA for total mesophilic bacteria, MRS for lactic acid bacteria, SB for enterococci, and BP for staphylococci and micrococci. Antibiotics utilized to supplement the culture media: Tc, tetracycline and Erm, erythromycin.

^b^Counts below the detection limit (2.0 log_10_⁡ cfu g^−1^).

∗Cheeses made from raw milk.

**Table 3:** Microbial counts (log_10_⁡ cfu g^−1^) of the total and antibiotic resistant bacterial populations found in samples of ten Italian cheeses.

Culture medium^a^	Cheese									
	Gorgonzola “dolce”	Gorgonzola “piccante”	Caprino∗	Quartirolo Lombardo	Pecorino Sardo∗	Grana Padano∗	Montasio∗	Monte Veronese∗	Asiago∗	Taleggio∗
PCMA	7.78	8.70	8.23	6.00	6.00	8.04	7.38	8.63	7.36	8.34
PCMA + Tc	5.04	4.48	3.00	2.05	<2.0^b^	3.60	<2.0	3.00	4.60	<2.0
PCMA + Erm	6.00	<2.0	<2.0	2.85	5.30	<2.0	<2.0	5.45	2.48	2.70

MRS	6.40	7.60	8.30	6.38	5.30	7.00	7.63	8.15	5.00	6.70
MRS + Tc	4.08	<2.0	<2.0	<2.0	<2.0	3.60	2.00	<2.0	3.67	<2.0
MRS + Erm	3.08	3.01	<2.0	<2.0	<2.0	<2.0	<2.0	<2.0	3.73	<2.0

SB	5.46	5.21	<2.0	6.00	4.88	3.60	4.41	3.20	5.30	2.00
SB + Tc	<2.0	<2.0	<2.0	2.30	<2.0	3.78	2.00	2.78	2.00	<2.0
SB + Erm	2.48	<2.0	<2.0	<2.0	2.00	<2.0	2.00	<2.0	<2.0	<2.0

BPA	5.95	5.44	<2.0	3.90	<2.0	3.97	2.60	3.78	<2.0	2.85
BP + Tc	3.78	2.48	<2.0	3.23	<2.0	3.60	2.00	<2.0	<2.0	<2.0
BP + Erm	3.00	<2.0	<2.0	2.48	<2.0	<2.0	<2.0	3.00	<2.0	<2.0

^a^Culture media used for counting of different bacteria groups: PCMA for total mesophilic bacteria, MRS for lactic acid bacteria, SB for enterococci, and BP for staphylococci and micrococci. Antibiotics utilized to supplement the culture media: Tc, tetracycline and Erm, erythromycin.

^b^Counts below the detection limit (2.0 log_10_⁡ cfu g^−1^).

∗Cheeses made from raw milk.

**Table 4:** Tetracycline and erythromycin resistance genes detected by conventional PCR in Spanish and Italian cheeses.

Cheese	Gene							
	Tetracycline resistance gene						Erythromycin resistance gene	
	*tet*(K)	*tet*(L)	*tet*(M)	*tet*(O)	*tet*(S)	*tet*(W)	*erm*(B)	*erm*(F)
Spanish cheeses								
Cabrales∗	+	+	+	−	+	+	+	−
Zamorano∗	+	+	+	−	+	+	+	−
Majorero∗	−	−	−	−	+	−	+	−
Mahón	−	−	−	−	−	+	−	−
Torta del Casar∗	−	−	+	+	+	+	+	−
Manchego	−	−	−	−	+	−	+	−
Ibores∗	−	−	+	−	+	+	+	−
Garrotxa	−	−	−	−	+	+	+	−
De La Mesta∗	+	+	+	+	+	+	+	+
Ibérico	−	−	+	−	+	−	+	−
Italian cheeses								
Gorgonzola “dolce”	−	−	−	−	−	−	+	−
Gorgonzola “piccante”	+	−	−	−	−	−	+	+
Caprino∗	−	−	−	+	+	+	+	+
Quartirolo Lombardo	−	−	−	−	+	+	−	−
Pecorino Sardo∗	+	−	+	−	+	+	−	−
Grana Padano∗	−	−	−	−	−	−	+	+
Montasio∗	−	−	−	−	−	−	+	+
Monte Veronese∗	−	−	−	−	+	−	+	+
Asiago∗	+	−	+	+	+	+	+	+
Taleggio∗	−	−	−	−	+	−	+	+

*Cheeses made from raw milk.
