# Supplementary material for: Molecular Identification and Quantification of Tetracycline and Erythromycin Resistance Genes in Spanish and Italian Retail Cheeses
Source: Biomed Res Int. 2014 Sep 11;2014:746859. doi: 10.1155/2014/746859 (PMC4180643; doi:10.1155/2014/746859)
Supplement: Supplementary file 1 — Supplementary Table 1 summarizes updated tetracycline and erythromycin resistance genes which were compiled for designing primers for the qPCR analyses. Nucleotide sequences were downloaded from the GenBank database and aligned with one another using Mega 5 Software. [file 746859.f1.docx]

**Supplementary Table 1.-** Resistance genes used to design primers utilized for the qPCR assays.

| **Resistance determinant** | **Species** | **GenBank**  **Accession no.** |
| --- | --- | --- |
|  |  |  |
| *tet*(M) | *Enterococcus faecalis* (Tn*916*) | U09422 |
|  | *Ureaplasma* sp. | U08812 |
|  | *Staphylococcus aureus* | AM990992 |
|  | *Clostridium difficile* | AJ5973138 |
|  | *Streptococcus pneumoniae* | FM211187 |
|  | *Klebsiella pneumoniae* | EU239355 |
|  | *Lactococcus lactis* subsp. *lactis* | DQ060147 |
|  | *Streptococcus pseudointermedius* | CP002478 |
|  | *Neisseria gonorrhoeae* | U58985 |
|  | *Escherichia coli* | JF830611 |
|  | *Listeria monocytogenes* | AJ704568 |
|  | *Pseudomonas* sp. | EF055282 |
|  |  |  |
| *tet*(W) | *Butyrivibrio fibrisolvens* | AJ222769 |
|  | *Faecalibacterium prausnitzii* | FP929045 |
|  | *Eubacterium siraeum* | FP929059 |
|  | *Bifidobacterium longum* | EU434756 |
|  | *Mitsuokella multacida* | AJ427422 |
|  | *Roseburia hominis* | CP003040 |
|  | *Bifidobacterium bifidum* | EU434755 |
|  | *Clostridium difficile* | AM749838 |
|  | *Streptococcus suis* | FN396365 |
|  | *Corynebacterium diphtheriae* | CP003209 |
|  | *Lactobacillus reuteri* | FJ489649 |
|  | *Lactobacillus acidophilus* | CP002559 |
|  |  |  |
| *tet*(S) | *Enterococcus faecium* | DQ95784 |
|  | *Enterococcus faecalis* | JN208881 |
|  | *Streptococcus thermophilus* | DQ377341 |
|  | *Listeria monocytogenes* | L09756 |
|  | *Lactococcus lactis* (pK214) | X92946 |
|  | *Lactobacillus plantarum* | AM039486 |
|  | *Enterococcus durans* | AM039488 |
|  | *Lactococcus garvieae* | NC010540 |
|  | *Streptococcus uberis* | EF092839 |
|  | *Streptococcus dysgalactiae* subsp. *equisimilis* | EF682209 |
|  |  |  |
| *tet*(O) | *Campylobacter jejuni* | M18896 |
|  | *Clostridium cellulovorans* | CP002160 |
|  | *Megasphaera elsdenii* | AY485123 |
|  | *Streptococcus pyogenes* | FR691055 |
|  | *Mobiluncus curtisii* | CP001992 |
|  | *Actinobacillus pleuropneumoniae* | AY987963 |
|  | *Pasteurella multocida* | FJ234438 |
|  | *Streptococcus pneumoniae* | FM178797 |
|  | *Enterococcus faecalis* | AY660532 |
|  | *Streptococcus suis* | CP002641 |
|  | *Clostridium butyricum* | GQ240297 |
|  |  |  |
| *tet*(L) | *Staphylococcus. aureus* | JN970906 |
|  | *Enterococcus faecium* | JF800907 |
|  | *Streptococcus gallolyticus* | FN597254 |
|  | *Geobacillus stearothermophilus* | M11036 |
|  | *Actinobacillus* sp. | AY359464 |
|  | *Streptococcus agalactiae* | X15669 |
|  | *Streptococcus pasteurianus* | AP012054 |
|  | *Streptococcus suis* | CP002644 |
|  | *Pediococcus* sp. | HQ651927 |
|  | *Escherichia coli* | GU584222 |
|  | *Pseudomonas* sp. | EF055266 |
|  | *Streptococcus epidermidis* | U35229 |
|  |  |  |
| *tet*(K) | *Staphylococcus aureus* | AB505629 |
|  |  |  |
| ermB | *Enterococcus faecalis* | M36722 |
|  | *Lactococcus garvieae* | AB290882 |
|  | *Enterococcus faecium* | HQ115078 |
|  | *Streptococcus pneumoniae* | AM490850 |
|  | *Streptococcus intermedius* | AF239772 |
|  | *Streptococcus suis* | CP002640 |
|  | *Lactobacillus plantarum* | FJ374272 |
|  | *Lactobacillus johnsonii* | DQ518904 |
|  |  |  |
| *ermF* | *Bacteroides fragilis* | M14730 |
|  |  |  |
